# India's death toll in 2021 during the COVID-19 pandemic: insights from delayed official civil registration data

**DOI:** 10.7189/jogh.15.03045

**Published:** 2025-11-14

**Authors:** Maxwell Salvatore, Brian Wahl, Bhramar Mukherjee

**Affiliations:** 1 Department of Biostatistics, Epidemiology, and Informatics, University of Pennsylvania Perelman School of Medicine, Philadelphia, Pennsylvania, USA; 2 Department of Genetics, University of Pennsylvania Perelman School of Medicine, Philadelphia, Pennsylvania, USA; 3 Department of Epidemiology of Microbial Diseases, Yale School of Public Health, New Haven, Connecticut, USA; 4 Yale School of Public Health, Yale Institute for Global Health, New Haven, Connecticut, USA; 5 Department of Biostatistics, Yale School of Public Health, New Haven, Connecticut, USA; 6 Department of Chronic Disease Epidemiology, Yale School of Public Health, New Haven, Connecticut, USA

## Abstract

India released its 2021 official death registration data in May 2025, showing a dramatic undercount of reported COVID-19 mortality in 2021 – a year that included the lethal second wave from the Delta variant in April to June 2021. The civil registration system (CRS) documented 10.2 million deaths in 2021 – a 26% increase from 2020 – compared to only 335 004 reported COVID-19 deaths. We calculated excess deaths as the difference between observed deaths and expected deaths had pre-pandemic mortality trends continued. Our analysis reveals India experienced approximately 2.4 million excess deaths in 2021, representing a 7.2× undercount compared to reported COVID-19 deaths. This aligns with the estimates derived from several epidemiological models during and after the pandemic, indicating excess-to-reported COVID-19 death ratios ranging from 4.4× to 11.9×. State-level analyses revealed considerable variation in reporting fidelity, with excess-to-COVID-19 death ratios ranging from under 2× in Kerala and Goa to over 40 in Gujarat. Limited disaggregated data showed excess death rates were substantially higher in men than women (2.2 *vs.* 1.3 per 1000 population), and in urban than rural areas (2.3 *vs.* 1.4 per 1000 population). Since the CRS data are incomplete in terms of age-stratified deaths, the proportional allocation of deaths by age according to external sources suggests excess death rates were higher in the 65 and older age group than in the population under 65 (14.1 *vs.* 0.9 per 1000 population). The four-year data delay and systematic underreporting underscore urgent needs for modernising India's surveillance system during acute phases of the pandemic and restructuring the official vital registration systems through protocol standardisation, real-time linkages, and infrastructure investments. Robust mortality tracking strengthens crisis preparedness and broader public health response.

Death registration systems worldwide faced unprecedented strain during the COVID-19 pandemic [1], exposing gaps in real-time mortality surveillance. Among the top 20 countries with the highest estimated excess deaths, excess-to-COVID-19 death ratios (EDR) range from 1.0× in the UK to 61.5× in Nigeria, with a global average of 2.7× [1]. Incomplete and inconsistent capture of the number of deaths during a public health crisis can hamper immediate pandemic response, prioritised resource allocation, and future preparedness. A case study of mortality reporting in India – home to nearly 18% of the global population – offers crucial lessons for strengthening vital statistics systems globally.

In May 2025, the Indian Ministry of Home Affairs released national civil registration system (CRS) data on births and deaths for 2021 [2]. These data provide an opportunity to assess the accuracy of reported COVID-19 deaths and real-time epidemiological estimates previously offered against the ‘ground truth’ of official records. Our exploration of national and state/union territory-level data on previously reported COVID-19-related deaths in 2021 from official sources; existing estimates offered by real-time epidemiological models; and the recently released official civil registration data highlights systemic weaknesses in mortality reporting and supports our short- and long-term recommendations. During acute phases of a pandemic, carefully designed surveys and surveillance tracking can provide rapid ascertainment of high-quality data on transmission and fatality rates of the circulating virus to ensure reliable input parameters into epidemic models, enabling realistic simulation scenarios that aid policymakers with real-time decision-making. These smart surveillance studies, alongside investments in improving the civil registration system’s completeness, accuracy, agility, and data granularity, are needed for India and many other countries with similar data struggles, enabling timely response and informing future crisis preparedness.

## MAGNITUDE OF UNDERREPORTING: WHAT THE DATA REVEALS

**Figure 1:**
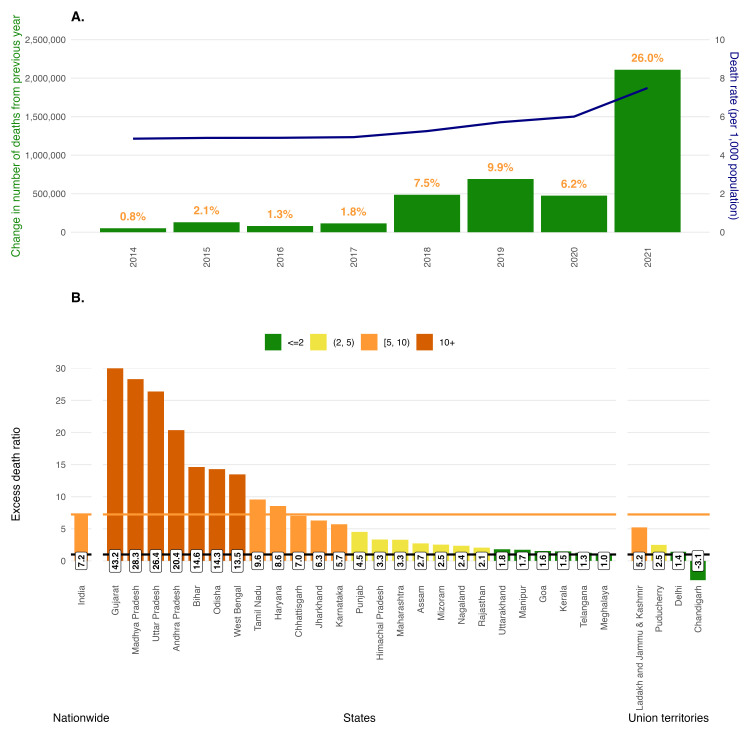
Registered deaths in India over time and excess-to-reported COVID-19 death ratios nationwide and by state/union territory. **Panel A.** Increase in deaths reported by the CRS over successive years in India from 2014 to 2021. The green bars represent the absolute difference in reported deaths in that year from the previous year. The orange numbers above the bars represent the percentage increase in deaths of the prior year. Death data comes from Statement 8 of the 2021 CRS report [4]. The blue line represents the crude death rate per 1000 over time, based on India’s 2019 National Population Commission population projections report [4]. **Panel B.** Ratio of excess deaths to COVID-19 reported deaths in India in 2021. We present this ratio nationwide and by state/union territory (restricting to those with more than 500 registered deaths in 2021). Excess deaths were estimated by applying the annual death rates observed in 2019 (CRS-reported deaths/2019 census-estimated population) to the 2021 census-estimated populations. The black horizontal line at 1.0 represents equal excess and COVID-19 deaths. The orange horizontal line represents the national excess-to-COVID-19 death ratio, 7.2. The y-axis is truncated at 30.

The May CRS report documents 10.2 million registered deaths in 2021 – a 26% relative increase from 2020, when there were 8.1 million registered deaths [3]. India reported a 6% relative increase in deaths from 2019 to 2020 (**Figure 1**, Panel A) [2]. The 2.1 million increase in registered deaths from 2020 to 2021 is ~ 6.3 times the reported 335 004 COVID-19 deaths in 2021 [5].

Excess death is a metric defined as the difference between observed deaths (as in the CRS report) and deaths that would have been expected if pre-pandemic mortality trends had continued. To calculate a simple estimate of expected deaths in 2021, we multiplied the 2021 population projection in India [4] by the 2019 crude annual death rate [2]. Based on this approach, we estimate India faced 2.4 million excess deaths in 2021 (Table S1 in the **Online Supplementary Document**) – implying an EDR of 7.2×. High EDR is a worldwide phenomenon – it is not unique to India. For example, the World Health Organization (WHO) estimated 14.8 million global excess deaths for 2020–21 compared to the reported 5.4 million COVID-19-related deaths – an EDR of 2.7× [1].

Sensitivity calculations using different baseline death rates from 2014–19 produce 2021 EDRs in India ranging from 7.2× to 10.7×, confirming substantial underreporting regardless of the choice of the baseline reference year (Table S1 in the **Online Supplementary Document**). These calculations assume consistent registration completeness and do not account for potential changes in the death registration rate during 2021. However, evidence suggests registration rates likely declined during the pandemic rather than increased [6–8], indicating the current estimate may be conservative.

## FOUR CRITICAL QUESTIONS FOR INDIA THAT INFORM GLOBAL HEALTH POLICY

The disconnect between real-time reporting of COVID-19 deaths and the released vital statistics report on the total number of deaths in 2021 poses four major questions:

1. How did reporting fidelity vary across geography and sociodemographic factors?
2. How accurate were epidemiological models in capturing this underreporting in the absence of observed death data as the pandemic was ongoing in 2021?
3. What proportion of excess deaths in India in 2021 can realistically be attributed to causes other than COVID-19?
4. How can one improve the capture of all-cause and cause-specific mortality data (during or beyond a pandemic) in a country of 1.4 billion people, where (based on 2021 estimates) 61% live in rural areas [4] and 47% of deaths happen outside a healthcare facility [2]?

## UNDERSTANDING VARIATION IN REPORTING FIDELITY

### State-level variations

State-level EDRs revealed dramatic variations in reporting fidelity: Gujarat had ~ 251 000 excess deaths, while reporting only 5800 COVID-19 deaths, a ratio of ~ 43× (**Figure 1**, Panel B). Three other states also demonstrated EDRs exceeding 20×, suggesting low reporting fidelity characterised by large populations, limited health infrastructure, and potential administrative barriers. Only six states had ratios below 2×, suggesting high reporting fidelity characterised by stronger health systems and better administrative capacity. Such discrepancies indicate weaknesses of real-time surveillance and timely death registration across a wide slate of jurisdictions.

State-level heterogeneity in EDRs reflects differences in COVID-19 underreporting, coupled with a confluence of multitude of factors such as: baseline differences in death registration completeness across states, classification convention of attributing cause of death as COVID-19 (such as deaths from COVID-19 *vs.* with COVID-19), health care system capacity and testing infrastructure, timing of pandemic waves, urban-rural population distribution, percentage of in-hospital mortality, active surveillance infrastructure and administrative efficiency. For example, the extreme EDR observed in Gujarat (>40×) likely reflects a cascade of factors listed above, including potential registration delays, an overwhelmed health care system during the severe second wave (April to June 2021), a large rural geography in the state, and possible data quality and reporting bias issues warranting further investigation. Though Basu and Adair [6] suggest that substantial state-level variation in death registration coverage is narrowing over time, the 2021 CRS report itself (chapter 2) highlights state-level variation in the adoption of registration guidelines, cadence of meetings, digitisation timelines and implementation, administration of defaulting penalties, completeness and frequency of registration unit inspection, and training of registration functionaries.

### Demographic patterns: by sex, residential location, and age

Using 2021 CRS report data, disaggregated by sex and residential location (rural/urban), coupled with stratified population projections and mortality rates from external sources, we calculated stratified crude and excess death rates (Table S1 in the **Online Supplementary Document**). As a proportion of total excess deaths, 64% occurred in males *vs.* females, and 53% in rural *vs.* urban areas. However, when normalised by the population size, the rate of excess deaths per 1000 was higher in males (2.2) compared to females (1.3), and higher in urban (2.3) areas compared to rural areas (1.4).

The CRS data provides incomplete age-stratified data, with some states/union territories absent and, among those providing data, some excluding delayed registered deaths, some reporting provisional data, and others reporting large quantities of deaths where age is not stated. Early studies during the pandemic in 2020 demonstrated disproportionate mortality impacts among younger populations, men, and marginalised communities in India [9,10]. Unlike in Western countries, where COVID-19 mortality was concentrated among the elderly, approximately 56% of COVID-19 deaths in India in 2021 were estimated to have occurred among those aged under 65 years [11]. In the absence of reported official data, we calculated age-stratified excess deaths based on nationwide excess death estimates and the proportion of age-stratified deaths reported by the 2024 UN Population Prospects [12]. In 2021, 93% of the Indian population was less than 65 years old [4]. When normalised by the population, the rate of excess deaths per 1000 was significantly higher in those aged 65 years and older (14.1) than in those under 65 years of age (10.9) (Table S2 in the **Online Supplementary Document**).

## EPIDEMIOLOGICAL MODELS *VS.* REAL-TIME DEATH REPORTING

India’s surveillance mechanisms captured only a fraction of COVID-19-related fatalities, while some epidemiological modelling approaches provided more realistic real-time estimates. Zimmermann and colleagues’ meta-analysis reported EDRs ranging from 4.4× to 11.9× in India, projecting 1.7–4.9 million COVID-19 deaths by June 2021, when reported numbers stood at 412 000 [13]. Institute for Health Metrics and Evaluation estimated 4.1 million excess deaths in India for 2020–21, the highest absolute excess mortality worldwide, corresponding with an EDR of 8.3× [14]. WHO estimated 4.7 million excess deaths in 2020 and 2021 compared to 481 000 reported COVID-19 deaths – an EDR of 9.8× [1]. Independent research from 12 states reported a 28% mortality increase during April 2020 to May 2021, projecting 3.8 million excess deaths [15].

In light of the data from the 2021 CRS report, it appears that compared to reported COVID-19 deaths, many real-time epidemiological models provided substantially more accurate estimates of the actual death toll. This highlights the vital contribution of careful real-time epidemiological modelling approaches during health emergencies in cases when surveillance systems fail or are incomplete. Countries should invest in robust surveillance systems *and* modelling infrastructure as complementary but critical approaches to pandemic monitoring.

## COVID-19’S CONTRIBUTION TO EXCESS MORTALITY

Excess deaths measure the overall death toll during a pandemic. They can be grouped into four categories: reported COVID-19 deaths, unreported/missed COVID-19 deaths, pandemic-related non-COVID-19 deaths, and long-term impacts, including long COVID-19 and increased risk for acute events (*e.g.* respiratory, cardiovascular) [16]. Globally, COVID-19 is overwhelmingly responsible for pandemic-era excess mortality. WHO analyses found that countries with low levels of COVID-19 transmission also reported low levels of excess deaths, suggesting that a high proportion of excess deaths are possibly attributable to COVID-19. the possible existence of an inverse association, as well [1]. A USA-based report quantified this proportion, concluding that roughly 66% of excess deaths in the country were attributable to COVID-19 [17]. Moreover, synchronous peaks in COVID-19 deaths and excess deaths suggest substantial underreporting and misclassification of COVID-19-realted deaths [18]. This auxiliary evidence indicates that India’s 2.4 million excess deaths in 2021 (or the 2.1 million reported in the Indian media [19], representing the difference between the total deaths in 2020 and 2021) largely reflect unrecognised COVID-19-related mortality.

Can this phenomenon be related to an increase in death registration rates in 2021? India’s Sample Registration System, a curated probability sample [20], has consistently found nearly 75–80% capture of registered deaths in India by the CRS in pre-pandemic years [6,7]. There is no reason to believe there has been a dramatic increase in death reporting during 2021 that can fully explain the excess deaths or attribute them to an increase in death registration rates. On the contrary, a survey suggested that almost 30% of deaths in India in 2019–21 were not captured by the civil registration system [8], meaning COVID-19’s true impact in India may likely exceed what the 2021 CRS report implies.

## LESSONS LEARNED AND RECOMMENDATIONS FOR RAPID DEATH REGISTRATION

Real-time reporting discrepancies and delayed official data releases lead to a singular conclusion: India must reimagine and restructure its pandemic surveillance strategies and its death registration system to be consistent and agile across states and union territories, while ensuring broad compliance with the Registration of Births and Deaths Act, 1969 [21]. It needs to enhance infrastructure in rural areas and incentivise nimble capture of cause-specific mortality. The 2021 CRS report notes that 47.3% of the deceased did not receive any medical attention at the time of death, which in part explains inadequate cause-of-death documentation [2]. This figure was reported to be 45.0% in 2020 [3], compared to 34.5% and 35.7% in 2019 [22] and 2018 [23], suggesting a pandemic-related increase in 2020 and 2021, possibly due to an overwhelmed health care system. The WHO civil registration strategic plan calls for continuous, universal, and timely recording of vital events through data transmission from local to central processing centres, enabling rapid crisis response [24]. This effort can leverage initiatives like the Ayushman Bharat Digital Mission [25] to allow nationwide morbidity and mortality data collection.

Crowd-sourced and innovative approaches for estimating excess deaths, like satellite imagery analysis of crematorium light intensity and tracking obituary rates compared to historical baselines in response to areas in northern India heavily affected during April to June 2021 [26,27], were commendable efforts to fill critical data gaps in real-time. However, no national infrastructure exists to produce and validate such estimates at this scale.

Real-time death estimation leveraging sampling-based study designs during a public health crisis, where a carefully chosen representative national/state sample of the population is followed through regular testing and outcome tracking, can better fill data gaps and inform inputs for epidemic modelling. For example, using National Family Health Survey and CVoter survey data, Jha and colleagues estimated 3.1–3.4 million COVID-19 deaths from June 2020 through July 2021 [15]. Other studies relying on subnational CRS data [28] and Consumer Pyramids Household Survey data [29] yield EDR estimates of around 8× (Table S3 in the **Online Supplementary Document**).

While the initial estimates remain inherently limited in scalability and reliability for systematic mortality surveillance, these innovative approaches can provide crucial information for epidemic models. In turn, simulation-based frameworks (*e.g.* BharatSim [30], InfluSim [31], Framework for Reconstructing Epidemic Dynamics [32], OutbreakTools [33]) can help policymakers to carry out sensitivity analysis and scenario-based projections where input parameters can be tweaked to allow for best- and worst-case situations and aid real-time decision-making. To be clear, such methods cannot substitute for the comprehensive national-level overhaul of vital statistics systems that India urgently needs.

How can countries like India build resilient mortality surveillance systems for accurate short- and long-term reporting? Based on these findings, we propose several recommendations to strengthen the health and mortality surveillance systems of any country struggling with tracking the number of deaths during a public health crisis (**Table 1**).

**Table 1:** Recommendations to strengthen health and mortality surveillance systems

Easily implementable	Long-term implementation
Development of small, carefully designed survey studies (*e.g.* adopting strategies from the Sample Registration System [20]) to establish real-time tracking and obtain high-quality severity and mortality data to inform input parameters for infectious disease models.	Digitisation and linkage of registration systems to rapidly share data across states/union territories (leveraging Ayushman Bharat Digital Mission [25]), and automate mortality reporting.
Building sophisticated modelling ecosystems that can leverage possibly imperfect and summary-level data to predict subnational outbreaks and simulate intervention impacts at a local level.	Increasing registration completeness to improve capture of out-of-hospital deaths by creating incentives for timely reporting.
Prioritising disaggregated data collection to identify subpopulations that are disproportionately affected and inform resource allocation.	Increasing data completeness and reducing missingness in elements like age, sex, and medically certified cause by adopting standardised protocols for death certification for all jurisdictions and linking hospital records with the civil registration system.
	Enhancing rural infrastructure investment and personnel training, equipping community health workers with the collection of digital mortality and morbidity data.

## CONCLUSIONS

We commend India for releasing these civil registration data and acknowledging the system's challenges. India’s experience reflects global challenges in pandemic mortality surveillance. While providing valuable retrospective insight, the four-year data delay demonstrates the urgent need to upgrade real-time vital statistics systems in India. This also provides some key lessons for other countries:

− Model-based estimates can provide reliable interim guidance when surveillance systems fail or are incomplete;
− State/region variations in reporting capacity require targeted interventions;
− Investment in vital statistics infrastructure is essential for health security and effective resource allocation;
− Transparency in data release, even when delayed, enables crucial learning.

We caution that our estimates are based on simple calculations and do not capture uncertainty. Many sophisticated methods for calculating expected deaths consider various factors and propagate and report uncertainty [14,34,35]; estimating expected mortality can be much more thorough than the simplistic approach we adopted [36–39]. Further, our calculations assume consistent registration completeness and do not account for potential variation across states in death registration during 2021. However, evidence suggests registration rates likely declined during the pandemic rather than improved [6–8], leading us to believe the reported numbers may be a conservative estimate of the total number of deaths. We additionally note that limited, incomplete disaggregated death data were available in the CRS, limiting our ability to make age-sex-urbanicity stratified calculations to contextualise our conclusions further using official data. Regardless, our simple calculations still provide a sense of the scale of underreporting.

India’s delayed 2021 mortality data reveal the magnitude of COVID-19’s true impact and the critical weaknesses in real-time mortality surveillance. The 7.2× undercount, while alarming, provides essential evidence for health systems strengthening. The path forward requires investments in digital infrastructure, standardised protocols, and enhanced capacity at the state and local levels. Most importantly, it demands political commitment to transparency and data quality as foundations of an effective public health response.

India’s experience offers a global roadmap for building more resilient vital statistics systems. The question is not whether future crises will occur, but whether countries will be prepared to count their true cost in real time.

## Additional material


Online Supplementary Document
